# A comparative analysis of the principal component analysis and entropy weight methods to establish the indexing measurement

**DOI:** 10.1371/journal.pone.0262261

**Published:** 2022-01-27

**Authors:** Robert M. X. Wu, Zhongwu Zhang, Wanjun Yan, Jianfeng Fan, Jinwen Gou, Bao Liu, Ergun Gide, Jeffrey Soar, Bo Shen, Syed Fazal-e-Hasan, Zengquan Liu, Peng Zhang, Peilin Wang, Xinxin Cui, Zhanfei Peng, Ya Wang

**Affiliations:** 1 School of Engineering and Technology, Central Queensland University, Sydney, Australia; 2 Shanxi Normal University, Xi’an, China; 3 Shanxi Fenxi Mining Zhongxing Coal Industry Co., Ltd, Lvliang, China; 4 School of Business, University of Southern Queensland, Ipswich, Australia; 5 GENEW Technologies Co. Ltd, ShenZhen, China; 6 Peter Faber Business School, Australian Catholic University, Blacktown, Australia; 7 Shanxi Kailain Technology Co. Ltd, Shanxi, China; 8 NetEase Inc., Hangzhou, China; University of Defence in Belgrade, SERBIA

## Abstract

**Background:**

As the world’s largest coal producer, China was accounted for about 46% of global coal production. Among present coal mining risks, methane gas (called gas in this paper) explosion or ignition in an underground mine remains ever-present. Although many techniques have been used, gas accidents associated with the complex elements of underground gassy mines need more robust monitoring or warning systems to identify risks. This paper aimed to determine which single method between the PCA and Entropy methods better establishes a responsive weighted indexing measurement to improve coal mining safety.

**Methods:**

Qualitative and quantitative mixed research methodologies were adopted for this research, including analysis of two case studies, correlation analysis, and comparative analysis. The literature reviewed the most-used multi-criteria decision making (MCDM) methods, including subjective methods and objective methods. The advantages and disadvantages of each MCDM method were briefly discussed. One more round literature review was conducted to search publications between 2017 and 2019 in CNKI. Followed two case studies, correlation analysis and comparative analysis were then conducted. Research ethics was approved by the Shanxi Coking Coal Group Research Committee.

**Results:**

The literature searched a total of 25,831publications and found that the PCA method was the predominant method adopted, and the Entropy method was the second most widely adopted method. Two weighting methods were compared using two case studies. For the comparative analysis of Case Study 1, the PCA method appeared to be more responsive than the Entropy. For Case Study 2, the Entropy method is more responsive than the PCA. As a result, both methods were adopted for different cases in the case study mine and finally deployed for user acceptance testing on 5 November 2020.

**Conclusions:**

The findings and suggestions were provided as further scopes for further research. This research indicated that no single method could be adopted as the better option for establishing indexing measurement in all cases. The practical implication suggests that comparative analysis should always be conducted on each case and determine the appropriate weighting method to the relevant case. This research recommended that the PCA method was a dimension reduction technique that could be handy for identifying the critical variables or factors and effectively used in hazard, risk, and emergency assessment. The PCA method might also be well-applied for developing predicting and forecasting systems as it was sensitive to outliers. The Entropy method might be suitable for all the cases requiring the MCDM. There is also a need to conduct further research to probe the causal reasons why the PCA and Entropy methods were applied to each case and not the other way round. This research found that the Entropy method provides higher accuracy than the PCA method. This research also found that the Entropy method demonstrated to assess the weights of the higher dimension dataset was higher sensitivity than the lower dimensions. Finally, the comprehensive analysis indicates a need to explore a more responsive method for establishing a weighted indexing measurement for warning applications in hazard, risk, and emergency assessments.

## Introduction

As the world’s largest coal producer, with the fourth largest coal reserves globally, China’s coal output remained at similar levels in 2020 as in 2019 at 3,690 MT, accounting for about 46% of global coal production [1, 2]. China spurred its economic recovery using coal from the lockdowns caused by the COVID-19 pandemic [2]. The underground coal mining industry continues to be an important sector for China’s economic development. Most coal seams are deep and require underground coal mining, accounting for about 60% of the world’s coal production [3]. Among present risks, methane gas (called gas in this paper) explosion or ignition in an underground mine remains ever-present [4]. A significant number (3,284) of coal mines have high gas content at outburst-prone risk levels across almost all 26 major coal mining provinces in China [5]. State Administration of China Coal Safety prevention regulations for Coal and Gas Outburst was updated on 1 October 2019 [6]. The majority of the current research mainly focused on exploring the methods and frameworks for avoiding reaching or exceeding the threshold limit value of the gas concentration from viewpoints of impacts on geological conditions and coal mining working-face elements [7]. Many techniques and methods were used to avoid reaching or exceeding the threshold limit value of the gas concentration. They included monitoring acoustic emission signals, electric radiation, gas emission, and micro-seismic on the physical properties of sound, electricity, and magnetism, thermal [8]. However, accidents associated with the complex elements of underground gassy mines need more robust monitoring and warning systems to identify risks, improving coal mining safety [9].

The research proposed that risks could be given a weighting factor for predictive control in monitoring the risk signals [10, 11]. In the literature, the weighted indexing measurement comprising the results of multi-criteria decision making (MCDM) to weighting critical factors enhanced safety warning indices. MCDM methods were significant for evaluating and ranking factors with conflicting characteristics in different fields and disciplines [12–15]. Some of them have been adopted to mapping the risks associated with natural hazards [16]. The outcomes to weighting factors can then represent and establish an indexing measurement [17]. The weighted indexing measurement can be adopted as a predictor for enhancing safety warning indices and building robust monitoring or early warning systems to improve coal mining safety. Literature divided MCDM methods into subjective methods, objective methods, and subjective and objective mixed methods. Many studies used either subjective methods or objective methods to weighting criteria. For reducing the potential bias of subjective or objective methods, more studies used subjective and objective-mixed methods to combine the advantages of different methods for weighting the criteria to build a weighted indexing measurement. However, real-time measurement with complex computing processes can significantly increase the system’s computational burden. Datasets are usually captured every 15 seconds or even less from the gas monitoring system in the coal mine industry. A real-time warning system to the coal mining applications cannot handle a complex MCDM method for implementation. Relatively limited attention has been paid to their appropriate selection for such decision problems [12], especially coal mine applications. There is a need to conduct studies for determining a single responsive approach to a safety warning system that may keep the computing system’s lower load.

This research focused on analyzing the most used objective methods–the PCA and Entropy- to avoid personal bias from the decision-makers. This research aimed to conduct a comparative analysis and determine which single method between the PCA and Entropy was better for establishing a responsive weighted indexing measurement to build robust monitoring or warning systems and improve coal mining safety. Qualitative and quantitative mixed research methodologies were adopted for this research, including analysis of two case studies, correlation analysis, and comparative analysis. The following section reports the literature review, the case studies, and comparative analysis, followed by conclusions and recommendations.

## Literature review

Literature divided MCDM methods into subjective methods, objective methods, and subjective and objective mixed methods. They depended on whether weight is calculated indirectly from the given methods, directly from the domain experts [15], or the decision-makers. Until 2019, 56 single and mixed MCDM methods were reported [12]. Each MCDM procedure has been developed with different advantages and disadvantages, though the scholars usually select an approach based on the nature and intricacy of the problem [18]. There are no criteria for the effectiveness of weighing methods [19]. The following section will discuss more used MCDM methods.

### Subjective weighting methods

Subjective weighting methods depend on the assessments of decision-makers. The design and determination of weights can be interpreted in terms of value judgments, that methods based on the subjective opinions of individual experts are preferred [19]. Decision-makers compare each criterion with other criteria and determine each pair of criteria [20]. They include Analytical Hierarchy Process (AHP), Best-Worst Method (BWM), Level Based Weight Assessment (LBWA), FUll Consistency Method (FUCOM). There are other less used methods: Conjoint method, Direct Point Allocation method, Decision Making Trial and Evaluation Laboratory (DEMATEL), Fuzzy Preference Programming (FPP), Linear programming, Measuring Attractiveness by Categorical Based Evaluation Technique (MACBETH), Multi-purpose Linear programming, proportional (ratio) method, Ratio or Direct Significance Weighting method, Resistance to Change method, simple Multi-Attribute Rating Technique (SMART), Step‐wise Weight Assessment Ratio Analysis (SWARA), Swing method, Tradeoff method, and Weighted Least Square (WLS) method [20].

The AHP method was used as the most common subjective method to determine the weights of the criteria/constructs in management problems [21, 22]. They assigned weights to the responses with the decision-makers choice [23]. The main advantage of AHP is used for determining the weights on top and bottom level criteria [14]. However, the AHP method needs to be performed in comparison in pairs of criteria [20]. The AHP method was almost impossible to perform entirely consistent comparisons in pairs with over nine criteria, which often overcame by dividing the criteria into subcriteria and would further make the model more complex [20]. Increasing the problem’s size will lead the decision-makers to meaningless pairwise comparisons among measures [14]. The AHP method is also limited to many comparisons as it needs to perform n(n-1)/2 comparisons in pairs of criteria [20].

The BWM is a newly established comparison-based method by Rezaei in 2015 [24, 25]. During the BWM process, experts were asked to select the best (most important) and worst (least important) criteria from each set of criteria: they were then asked to perform pairwise comparisons between the criteria using a number between 1 and 9 [25]. Also, this method used an equation to calculate the consistency ratio for verifying the validity of comparisons [26]. The BWM technique can be applied to efficiently and reliably solve multi-criteria problems [27]. BWM has the following advantages over other methods of requiring the use of fewer comparison data and therefore has better consistency in pairwise comparisons, achieving more reliable weight results, being easy to understand and revise by decision-makers for increased consistency [20, 24, 27] as: 1) being in the smaller number of pair comparisons (2n-3); 2) using fewer pairwise comparison matrices thereby less time to implement; having better consistency than existing subjective weighting methods, and: requiring less comparative data. However, this model is unacceptable to many researchers as many comparisons in pairs of criteria, defining the limitations for solving non-linear models, and solving non-linear models make the application of the BWM significantly more complex [20].

The LBWA method was developed to meet the need for a process whose algorithm requires a few comparisons in pairs of criteria and has a rational and logical-mathematical algorithm [20]. The advantages of the LBWA model were outlined by the recent studies [20, 28] as: being suitable for use in complex models with a larger number of evaluation criteria; not being more complicated with the increase of the number of criteria; allowing the calculation of weight coefficients with the small number of criteria comparisons; being the flexibility to enable decision-makers to present their preferences and eliminate inconsistencies through logical algorithm when prioritizing criteria, and; not being limited to integer values from the predefined scale.

The FUCOM method was developed by [29] to determine the weights of criteria [30]. The main advantage of the methodological procedure of FUCOM eliminates the problem of redundancy of pairwise comparison, which is present in some subjective models for determining the weight of the criteria [31]. FUCOM requires only (n-1) pair-wise Comparisons for n criteria against n (n − 1)/2 comparisons in the case of AHP [21] and (2n-3) in the case of BWM [20]. FUCOM is also that when the relation between consistency and the required number of criteria comparisons is considered, this method provides better results than the AHP and BWM methods [29]. The main limitation of FUCOM is the lack of more studies to verify the validation of this model due to the literature review.

### Objective weighting methods

The objective method determines the weight-based known evaluation information by solving a mathematical model, which is particularly useful in situations where the decision-makers do not exist, or the options of the decision-maker are inconsistent [15]. Several objective methods have mainly been discussed in the literature, including Criteria significance Through Intercriteria Correlation (CRITIC), Entropy, FANMA, The Principal Component Analysis (PCA), and Technique for Order Preference by Similarity to Ideal Solution (TOPSIS). The CRITIC approach is one of the essential weighting models to estimate the objective weights of the attributes [15, 18]. This method used the standard deviation and the correlation coefficient between the criterion and other criteria to quantify the value of each feature and compute the attribute weights [15, 18].

The Entropy method was proposed by Shannon & Weaver in 1947 and further to be emphasized by Zeleny in 1982 [23]. The entropy method was currently used as the most common objective method to determine the weights of the criteria/constructs [22]. The Entropy established the objective weights for the attributes/responses: defining the importance of every response but not include any thoughtfulness of the preference of the decision-makers [23]. The Entropy measures the uncertainty of variables and evaluates how the controlling factors influence the outcome [16]. It delivers a quantitative measure of information content that can compare and analyze the effect of using different statistical models, algorithms, and corresponding tuning parameters [32] and believes that the lower the entropy of the criterion, the more valuable information the criterion contains [19]. This method has been highly influential in modeling and mapping different natural hazards [16].

The FANMA method was derived from the names of the authors of the technique by [33]. It was currently lack reported in the literature rather than in Serbian. Therefore, the FANMA method was discussed in this manuscript. The PCA method is a dimension reduction technique to transform a high-dimensional dataset into a low-dimensional one while preserving the information content [34, 35]. It distilled multiple, potentially correlated variables into new, independent constructs/factors; typically, the number of constructs is much smaller than the number of variables in the original data set [34]. PCA can be handy for identifying the most critical variables or the main contributing factors to the phenomenon based on the common factors under investigation and conclude the linear relationship between variables by extracting the most relevant information in the dataset [34, 36].

The TOPSIS method is a classical tool used to solve MCDM [37]. In the literature, the TOPSIS method has been another broadly used MCDM approach since 2020. TOPSIS technique is straightforward to construct the problem, easily understandable, and demonstrating adequate computational efficiency, which provides a scalar value that accounts for both best and worst alternatives’ ability to measure the relative performance for each choice in a simple mathematical form [14]. The TOPSIS method allows the weighting of each criterion from the decision-maker regardless of the level [14]. The disadvantage of TOPSIS is that for the criteria for which higher values were preferred, the larger the criterion outcomes were, the greater were the preferences attached [38]. Another disadvantage is that the adoption of TOPSIS needs to consider two problems—the rank reversal and heterogeneity of the criteria [39].

### Subjective and objective-mixed weighting methods

Based on the above briefly discussions, both the objective and subjective methods had limitations and disadvantages. For reducing the potential bias of subjective or objective methods, many studies used subjective and objective-mixed methods to combine the advantages of different methods for weighting the criteria to build a weighted indexing measurement. It is assumed that the combined weighting method will reduce the potential bias of a single subjective or objective weight or make up for the deficiency of subjective [19].

For example, Lin, Pan & Chen (2020) used an Entropy and TOPSIS mixed method to evaluate Urban air quality, which used the entropy to calculate the weights and then used the TOPSIS method to measure the air quality level [40]. Subba et al. (2020) studied quality criteria for groundwater use through the Entropy index and PCA method, which used the Entropy index to quantify water quality and use the PCA method to explain the interrelated variance variables dimensionality of the datasets [41]. Tan & Zhang (2020) studied a decision-making method based on Entropy to typhoon disaster assessment and used the evaluation based on distance from the average solution (EDAS) method to rank and select the best alternative [42]. Zhang et al. (2020) proposed a safety assessment in road construction work systems based on an AHP and PCA mixed method that used a group AHP to collect experts’ views on these indicators’ relative importance and then used PCA to create a low-dispersion group judgment matrix [43]. Tahir & Zeeshan (2021) proposed a novel TOPSIS method based on the Entropy measure to solve the multi-attribute decision-making problems [37]. Teixeira et al. (2021) used Entropy to determine the weights of various constructs/criteria and support the AHP [22]. Wang et al. (2021) used an Entropy and TOPSIS mixed method to evaluate agricultural extension service for sustainable agricultural development [44]. The TOPSIS method was be applied for ranking, while the Entropy method was used to determine the weight with good stability [44]. Wei et al. (2021) used the Entropy and AHP methods for karst collapse susceptibility assessment, which used AHP to build the structure model according to experts’ judgment [45]. They then combined the catastrophe theory to calculate a weight-based only on the observation data without considering subjectivity.

However, real-time measurement and evaluation of complex functions and processes can significantly increase the computational burden for implementation [46]. Datasets are usually captured every 15 seconds or even less from the gas monitoring system in the coal mine industry. A real-time warning system to the coal mining applications cannot handle a complex MCDM method for implementation. Relatively limited attention has been paid to their appropriate selection for such decision problems [12], especially coal mine applications. There is a need to conduct studies for determining a single responsive approach to a safety warning system that may keep the computing system’s lower load.

### The selection of MCDM methods

Regardless of the MCDM method of choice, the common goal is to select and evaluate available alternatives and determine the weights based on a large number and variety of criteria [19, 27]. The selection of an MCDA should be suitable for solving a specific decision problem and the research questions on the particular study area characteristics [12, 16]. The selected MCDM methods may screen, prioritize, sort, or select a series of alternatives under commonly disproportionate, independent, or conflicting criteria and rely on calculating measures (attribute) weight [15]. A higher weight may prioritize the performance indicator [19]. Thus, selecting the proper MCDA method became a vital element of MCDM in decision-making [12, 13, 47].

Up-to-date literature highlighted that subjective weighting methods did depend on the assessments of decision-makers and had at least two limitations. The main limitation is that the weighted analysis results or the ranking of the alternatives will be impacted by the level of knowledge of the domain experts in the related domains since the criteria weight determined by the subjective method denotes the decision-maker’s personal information [15]. In actual decision-making situations, improper human judgments raise the level of vagueness [28]. Another limitation is that a large number of comparisons makes the application of the model more complex, especially in cases of a large number of criteria [20]. Comparing to subject methods, objective methods combined the strength comparison of each criterion with the conflict between the criteria [15]. Subjective methods were excluded from this research’s scope and not be discussed in the following sections.

The literature review searched publications between 2017 and 2019 in CNKI—the largest and most continuously updated Chinese journal database [48]. A total of 25,831 studies were searched on the subject methods. Seven methods were the most used in China without any decision-maker’s choices, including Artificial Neural Networks (ANN), Coefficient of Variation (CoV), CRITIC, Entropy, PCA, Rough Sets, and TOPSIS [49, 50]. However, no study reported which method was most used to hazard, risk, and emergency assessment. Results indicated the PCA (41.81% in total, 10,801 out of 25,831; 41.38% in 2017, 3,329 out of 8,045; 42.07% in 2018, 3,628 out of 8,624; and 41.96% in 2019, 3,844 out of 9,162) as the predominant method adopted (Table 1). Entropy is the second most widely adopted method (15.80% at total, 10,801 out of 25,831; 15.16% in 2017, 1,220 out of 8,045; 15.46% in 2018, 1,333 out of 8,624; and 16.69% in 2019, 1,529 out of 9,162). TOPSIS (15.23%, 3,935 out of 25,831), Rough Sets (13.48%, 3,481 out of 25,831), ANN (9.10%, 2.351 out of 25,831), and CoV (4.57%, 1,181 out of 25,831) were followed. The CRITIC method (0.81%, 209 out of 25,831) was the least used approach.

**Table 1:** Objective methods in publications between 2017 and 2019.

Methods	2017	%	2018	%	2019	%	Sum	%
**PCA**	3,329	41.38%	3,628	42.07%	3,844	41.96%	10,801	41.81%
**Entropy**	1,220	15.16%	1,333	15.46%	1,529	16.69%	4,082	15.80%
**TOPSIS**	1,120	13.92%	1,294	15.00%	1,521	16.60%	3,935	15.23%
**Rough Sets**	1,167	14.51%	1,227	14.23%	1,087	11.86%	3,481	13.48%
**ANN**	830	10.32%	767	8.89%	754	8.23%	2,351	9.10%
**CoV**	379	4.71%	375	4.35%	427	4.66%	1,181	4.57%
**CRITIC**	50	0.62%	74	0.86%	85	0.93%	209	0.81%
**Total**	8,045	100.00%	8,624	100.00%	9,162	100.00%	25,831	100.00%

The following section focused on determining which single method between the PCA and Entropy methods will be the best choice for establishing the indexing measurement to the coal mine applications.

## Research methodology

**Fig 1:**
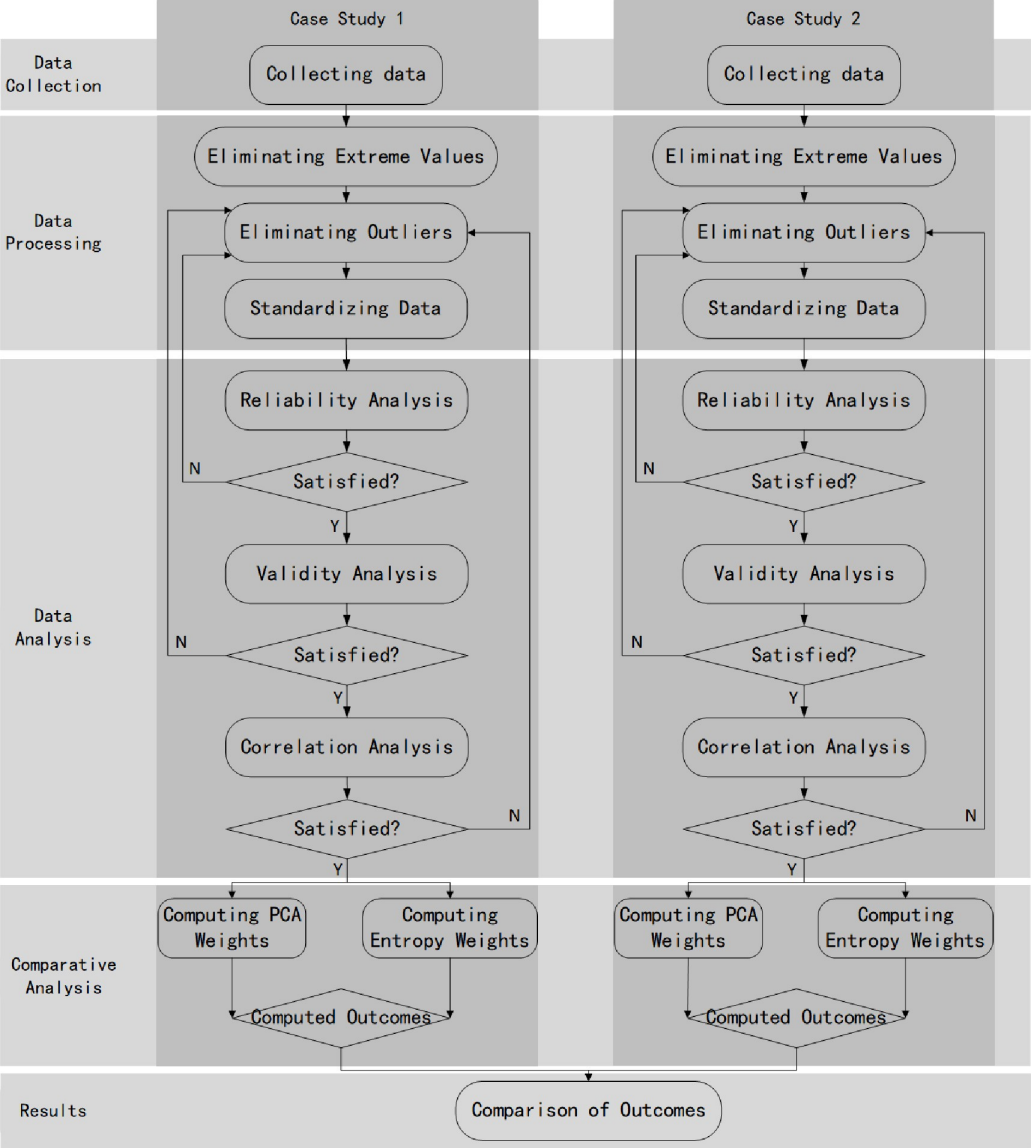
The research procedure flowchart.

The qualitative and quantitative mixed research methodology was adopted in this research, including two case studies, correlational research and comparative analysis. Fig 1 shows the research procedure processes of this study. It started with data collection, data processing, data analysis, comparative analysis, and outcomes. During data collection, data were obtained from two Case Study mines separately.

The data preprocess consists of transforming the data values of a specific dataset, aiming to optimize the information acquisition and process. As there is a contrast between the maximum and minimum values of the dataset, normalizing the data minimizes the algorithm’s complexity for its corresponding processing [51]. Data processing covered three procedures: eliminating extreme values, eliminating outliers, and standardizing data. The extreme data value (also called extreme value in this paper) can substantially bias inference [52]. The box-plot technique was adopted for data cleaning procedures. It was a better tool for responding to variation in generalized extreme value shape parameters [53] and a simple way of commonly employing a resistant rule to identify possible outliers in a single batch of the univariate dataset [53].

Data standardization was then followed as data were collected from the different sensors with a variety of measurements. The most common standardizing data approaches include z-score normalization, min-max standardization, distance to target normalization, and ranking normalization [51, 54]. The Z-score normalization approach will be used in this research. The reason is that a data standardization based on the scaling of variables using the z-score algorithm may increase the outcomes’ precision compared to other techniques [51].

A correlational research method was adopted in data analysis, comprising reliability analysis, validity analysis, and correlation analysis. As a quantitative research method, the correlational research method was adopted to measure two variables and assess the statistical relationship (i.e., the correlation) between them with little or no effort to control extraneous variables [55]. When the correlational research method is adopted, correlation analysis will confirm a strong relationship between the data. Correlation analysis can find comprehensive results to find a linear relationship between linear-dependent variables if it exists: it can give a solid indicator to interpret a robust nonlinear relationship between nonlinear-dependent variables [56].

The comparative analysis then followed. Both the PCA and Entropy methods were then computed separately for two Case Studies. The computed outcomes determined which weighting factor method would be a single responsive approach for the case choice. An SPSS statistic package version 26 was used for conducting data analysis in this research.

## Case studies

Case Study 1 obtained data between 00:00:00 am on 16 December 2019, and 5:31:00 am on 19 December 2019 from mine No.1209 at Shanxi Fenxi Mining ZhongXing Coal Industry Co. Ltd (ZhongXing)—a large coal company in China. Case Study 2 used data between 00:00:00 am on 25 September 2020 and 20:48:00 pm on 16 October 20220 from Mine No.4 North at ZhongXing.

### Data collection in Case Study 1

**Fig 2:**
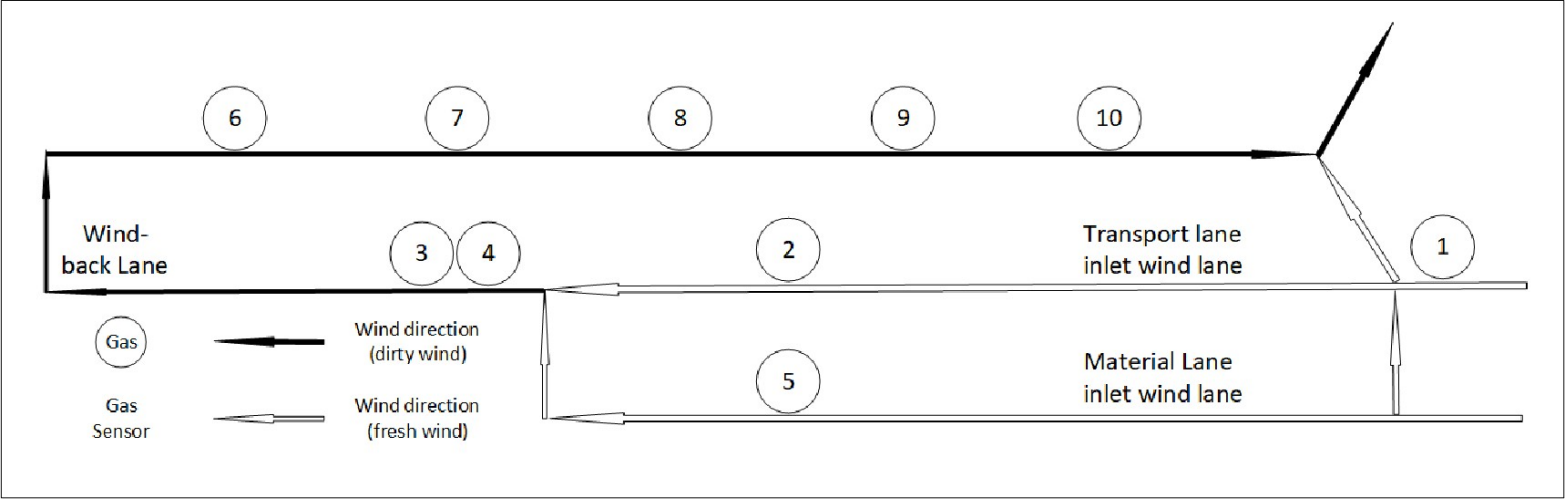
Ten gas sensors installed in Case Study mine 1.

Ten gas sensors were installed in Case Study mine 1 [7] (Fig 2). Table 2 shows the codes of the sensors used. This research initially obtained 17,280 data outputs from each gas sensor. The box-plot technique was adopted to eliminate the extreme values and outliers. The final data was 7,265 for each gas sensor. Thus, 72,650 datasets in total were collected for ten sensors (S2 Appendix).

**Table 2:** Code of sensors in Case Study mine 1.

No.	Sensor Name	Code
**1**	Coal Bin T	T030601
**2**	Transport Lane T	T030602
**3**	Working Face T	T030603
**4**	Upper Corner T	T030604
**5**	Material Lane T	T030701
**6**	1000m Refuge Chambers T	T030801
**7**	middle of Wind-back Lane T	T030802
**8**	500m Refuge Chambers T	T030803
**9**	Wind-back Lane T	T030804
**10**	Wind-back Lane Mixing T	T030805

### Data collection in Case Study 2

**Fig 3:**
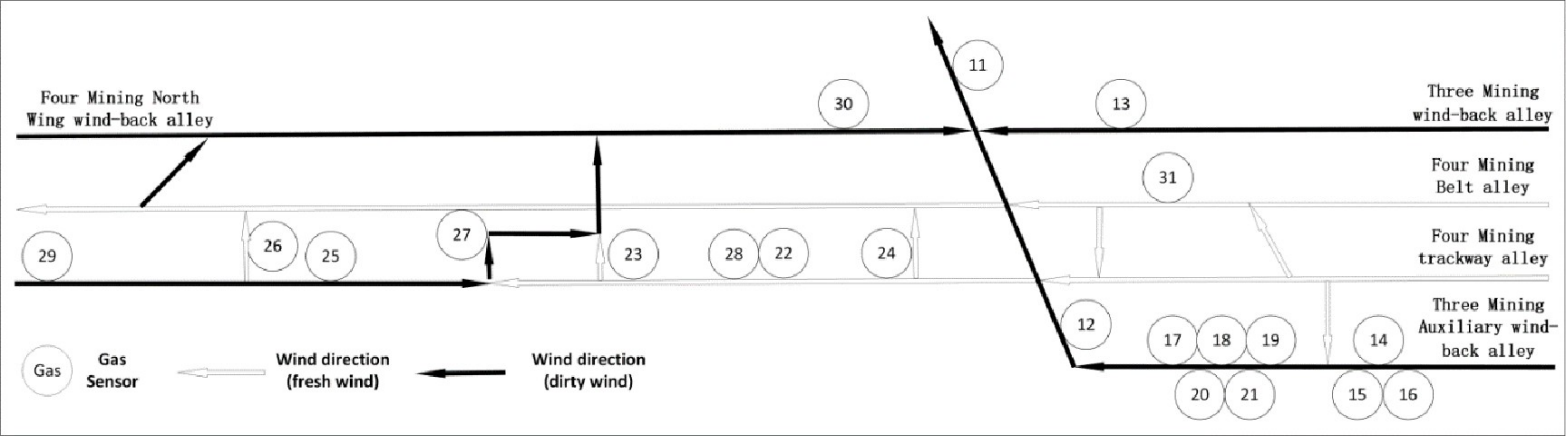
21 gas sensors installed in Case Study mine 2.

Twenty-one gas sensors were installed in Case Study mine 2 (Fig 3). Table 3 shows the codes of the sensors used. This research initially obtained 65,535 data outputs from each gas sensor. 1,376,235 data were obtained. The box-plot technique was used to eliminate the extreme values and outliers. The final data was 9,430 for each gas sensor and 198,030 in total (S3 Appendix).

**Table 3:** Code of sensors in Case Study mine 1.

No.	Sensor Name	Code
**1**	Three mining Total wind-back alley T	T010101
**2**	Three mining auxiliary wind-back alley T	T010102
**3**	Three mining East Wing wind-back alley T	T010103
**4**	Three mining Emergency Shelter Back Transition Room T	T010104
**5**	Three mining Emergency Shelter Front Transition Room T	T010105
**6**	Three mining Emergency Refuge Survival Room T	T010106
**7**	Four mining water bin working face T	T010201
**8**	Four mining water bin wind-back alley T	T010202
**9**	Four mining water bin air vent T	T010203
**10**	Four mining water bin fan front T	T010204
**11**	Four mining water bin Mixing T	T010205
**12**	Four mining trackway 500m Refuge Chambers T	T010301
**13**	Four mining trackway air vent T	T010302
**14**	Four mining trackway fan front T	T010303
**15**	Four mining trackway working face T	T010304
**16**	Four mining trackway wind-back alley T	T010305
**17**	Four mining trackway Mixing T	T010306
**18**	Four mining trackway middle T	T010307
**19**	Four mining trackway Downwind side of the rig T	T010308
**20**	Four mining North Wing wind-back alley T	T010401
**21**	Four mining belt lanes Coal Bin T	T010501

## Data analysis in Case Study 1

### Reliability and validity analysis

An SPSS statistic package version 26 was used for data analysis. P values of 0.05 are considered as the ’gold standard’ of significance [57]. For building the safety warning indices, however, this research considered that the smaller the significance value, the lower the risk of rejecting the null hypothesis when it is true. Therefore adopted 0.01 as a significance level. Ten items were delivered for the reliability analysis. Six items were rejected (T030601, T030602, T030603, T030604, T030701, and T030801) due to dissatisfaction with the value of Cronbach’s Alpha (lower than 0.6). Four items (T030802, T030803, T030804, and T030805) remained. Through the reliability analysis, convergent validity was also established. Convergent validity was assessed through reliability analysis in this research; other validity tests were also conducted.

The final results indicated that the remaining four items satisfied an exploratory study’s reliability analysis requirements and validity standards, listed in Table 4. Cronbach’s Alpha was 0.968 and was considered to have acceptable reliability (above 0.8). The ratio of the number of cases to variables was 1816:1 (greater than 5:1). Kaiser-Meyer-Olkin (KMO) was 0.823 and considered to have an acceptable measure (greater than 0.8). Bartlett’s test of sphericity was 0.000 (p<0.001). All communalities after extraction were greater than 0.50. The average communality was 0.957 (greater than 0.6). All anti-image correlations were satisfied (above 0.5).

**Table 4:** Reliability and validity analysis of gas data in Case Study 1.

**Descriptive Statistics**				**Communalities**		**Cronbach’s Alpha**	**Validity Analysis**		
	Mean	Std. Deviation	Analysis N	Initial	Extraction		Kaiser-Meyer-Olkin Measure of Sampling Adequacy.		.823
**T030802**	.3084	.03641	7265	1.000	.884	.968			
**T030803**	.3688	.03886	7265	1.000	.912		Bartlett’s Test of Sphericity	Approx. Chi-Square	51040.029
**T030804**	.3641	.03838	7265	1.000	.968			df	6
								Sig.	.000
**T030805**	.2792	.02730	7265	1.000	.956		Average Communality		.957
							Anti-image Correlations		>0.5

### Correlation analysis

A correlation analysis was followed to undertake and confirm whether there was a strong relationship between items. The Pearson product-moment correlation coefficient (called correlation coefficient in this research) is a measure used to describe the linear association between two random variables [58]. The correlation coefficient helps assess the linear correlation or relationship between two samples: the degree of fit between two samples of interest is given in the correlation coefficient; a value approaching unity indicates a robust linear relationship and vice versa [59].

SPSS statistic package version 26 was used to conduct Pearson correlation analysis. The results showed that correlation coefficients were 0.862 between T030802 and T030803, 0.894 between T030802 and T030804, and 0.873 between T030802 and T030805. Correlation coefficients were 0.862 between T030803 and T030802, 0.912 between T030803 and T030804, and 0.908 between T030802 and T030805. Correlation coefficients were 0.8894 between T030804 and T030802, 0.912 between T030804 and T030803, and 0.985 between T030804 and T030805. Correlation coefficients were 0.873 between T030805 and T030802, 0.908 between T030805 and T030803, and 0.985 between T030802 and T030804. All correlation coefficients indicated that robust correlations (greater than 0.3) existed between every two items at the 0.01 level (Table 5).

**Table 5:** Correlation analysis in Case study 1.

Correlations					
		T030802	T030803	T030804	T030805
**T030802**	Pearson Correlation	1	.862**	.894**	.873**
	Sig. (2-tailed)		0.000	0.000	0.000
	Sum of Squares and Cross-products	9.629	8.860	9.074	6.305
	Covariance	.001	.001	.001	.001
	N	7265	7265	7265	7265
**T030803**	Pearson Correlation	.862**	1	.912**	.908**
	Sig. (2-tailed)	0.000		0.000	0.000
	Sum of Squares and Cross-products	8.860	10.969	9.886	7.000
	Covariance	.001	.002	.001	.001
	N	7265	7265	7265	7265
**T030804**	Pearson Correlation	.894**	.912**	1	.985**
	Sig. (2-tailed)	0.000	0.000		0.000
	Sum of Squares and Cross-products	9.074	9.886	10.703	7.500
	Covariance	.001	.001	.001	.001
	N	7265	7265	7265	7265
**T030805**	Pearson Correlation	.873**	.908**	.985**	1
	Sig. (2-tailed)	0.000	0.000	0.000	
	Sum of Squares and Cross-products	6.305	7.000	7.500	5.413
	Covariance	.001	.001	.001	.001
	N	7265	7265	7265	7265

**. Correlation is significant at the 0.01 level (2-tailed).

### Computing the weights

The main idea of PCA is to analyze the characteristic properties of a covariance matrix to obtain the principal components of data (eigenvectors) and their weights (eigenvalues) by retaining the lower-order principal component (corresponding to the maximum eigenvalue) [60]. The detailed processes of PCA have been discussed widely. This research used an SPSS analysis package and an Excel sheet to compute the weighting results, which followed the PCA algorithm/procedure of a recent study by [60].

The processes of Entropy included first deciding objectives (decision matrix) and then calculations of the normalized decision matrix, probability of the attribute/response to take place, the entropy value of attribute/response, and degrees of divergence (average information contained) by each response and after that entropy weight [61]. In this research, the Entropy algorithm followed the step-by-step weight estimation by [19] as:

Nomenclature:

a_ij_: elements of decision matrix (DM)

r_ij_: normalized elements of decision matrix

w_j_: weight or importance of criteria (j = 1,…, n)

The intensity (p_ij_) of the j-th attribute of the i-th alternative is calculated for each criterion (Sum-method):

$$p_{ij}=\frac{r_{ij}}{\sum_{i=1}^{m}r_{ij}},\forall_{i}=1,\ldots ,m,j=1,\ldots ,n;\sum_{i=1}^{m}p_{ij}=1$$ (1)

To calculate the entropy (ej) and the key indicator (qj) of each criterion:

$$e_{j}=-\frac{1}{\ln m}\cdot \sum_{i=1}^{m}p_{ij}\cdot \ln p_{ij},j=1,\ldots ,n;\;(if\;p_{ij}=0\Rightarrow p_{ij}\cdot \ln p_{ij}=0)$$ (2)

$$q_{j}=1-e_{j},\;j=1,\ldots ,n$$ (3)

To calculate the weight of each criterion:

$$w_{j}=\frac{q_{j}}{\sum_{k=1}^{n}q_{k}},j=1,\ldots ,n$$ (4)

The open-source code for running the above Entropy algorithm was provided [7] (S1 Appendix).

**Fig 4:**
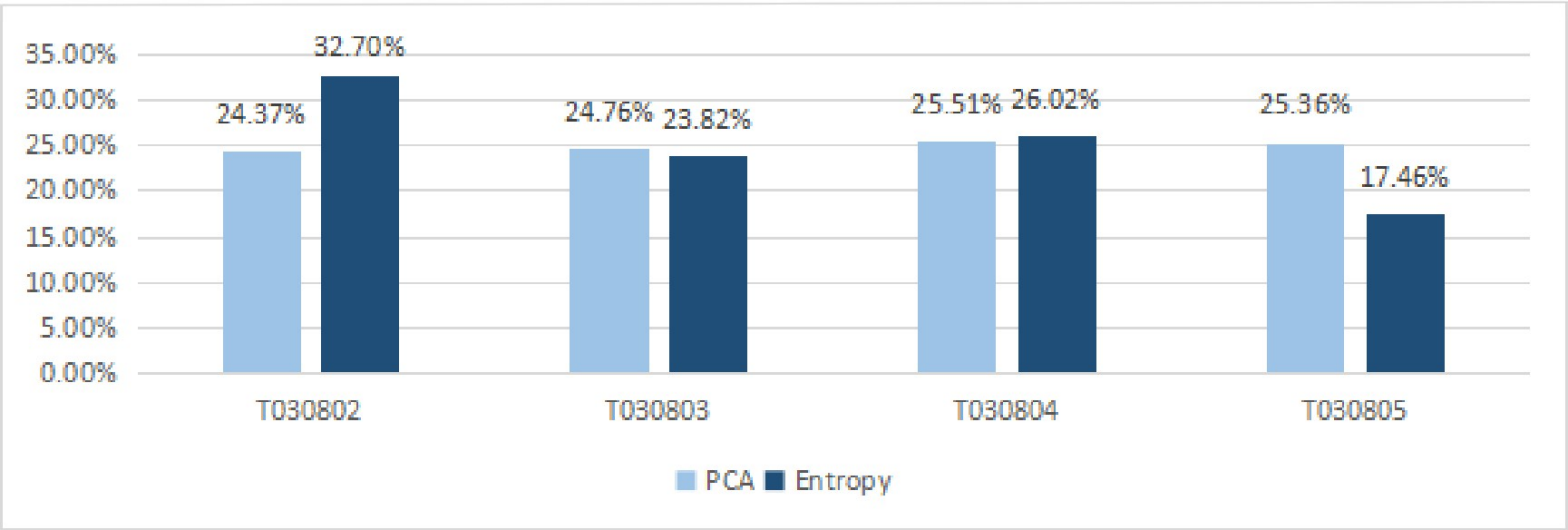
The computed outcomes of the weights in Case Study 1.

The PCA and Entropy were then computed separately to determine which weighting factor method would be a single responsive approach for the case 1 choice. The values of the PCA and Entropy on T030802, T030803, T030804 and T030805 were 24.37% and 32.70%, 24.76% and 23.82%, 25.51% and 26.02%, and 25.36% and 17.46%, respectively (Fig 4).

Table 6 describes the mean and standard deviation statistics for the PCA and Entropy methods adopted to Case Study 1. For the weights to T030802, the minimum value is 0.2437; the maximum value is 0.327; the mean value is 0.2854; the standard deviation is 0.0059. For the weights to T030803, the minimum value is 0.2382; the maximum value is 0.2476; the mean value is 0.2429; the standard deviation is 0.0007. For the weights to T030804, the minimum value is 0.2551; the maximum value is 0.2602; the mean value is 0.2577; the standard deviation is 0.0004. For the weights to T030805, the minimum value is 0.1746; the maximum value is 0.2536; the mean value is 0.2141; the standard deviation is 0.0056. It means that all data are relatively distributed near the mean value.

**Table 6:** Descriptive on standard deviation to Case Study 1.

	Entropy	PCA	N	Mean	Std. Deviation
**T030802**	32.70%	24.37%	2	28.54%	0.0059
**T030803**	23.82%	24.76%	2	24.29%	0.0007
**T030804**	26.02%	25.51%	2	25.77%	0.0004
**T030805**	17.46%	25.36%	2	21.41%	0.0056
**Valid N (listwise)**			2		

**Fig 5:**
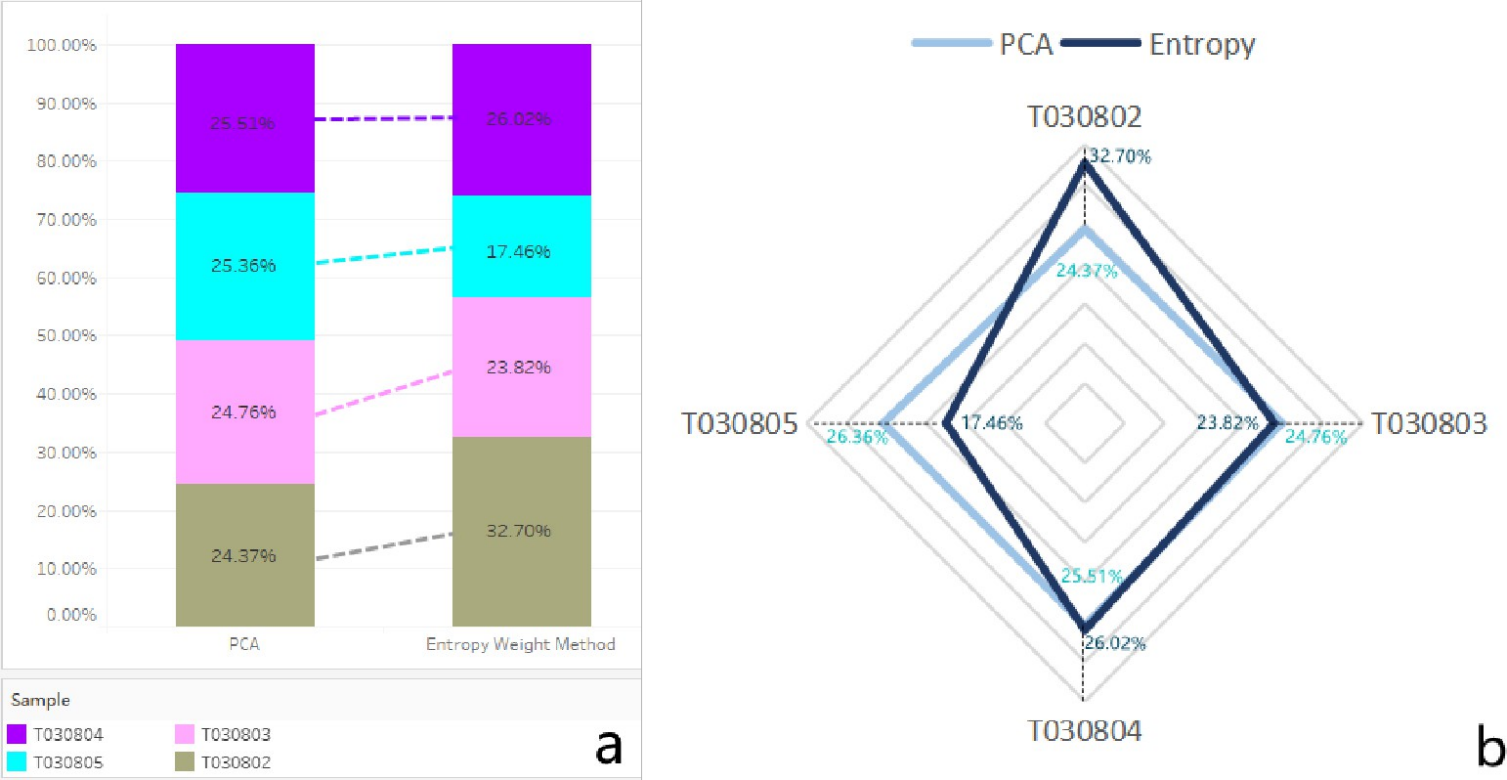
Visualization of comparison of the weighted index in Case Study 1.

The comparisons of the weighting methods between the PCA and Entropy methods were demonstrated in Fig 5. The demonstration clearly shows the different values of the two methods. The results were visually set to a weighted indexing measurement with a radar visualization for The PCA (with blue color) and Entropy (with deep blue color) weighted index (Fig 5B).

## Data analysis in Case Study 2

### Reliability and validity analysis

Data from five gas sensors were rejected during the reliability analysis due to dissatisfaction with the value of Cronbach’s Alpha (lower than 0.6), including T010101, T010102, T010204, T010301, and T010302. Convergent validity was established through reliability analysis in this research. Other validity tests were then conducted for the remaining sixteen items.

The results indicated that the remaining items have strong evidence of meeting the reliability analysis, validity analysis, and correlation standards of the exploratory research as listed in Table 7. Cronbach’s Alpha was 0.895 and was considered to have acceptable reliability (above 0.8). The ratio of the number of cases to variables was 589:1 (greater than 5:1). KMO was 0.904 and considered to be acceptable (greater than 0.8). Bartlett’s test of sphericity was 0.000 (p<0.001). The average communality was 0.701 (greater than 0.6). All anti-image correlations were satisfied (above 0.5).

**Table 7:** Reliability and validity analysis of gas data in Case Study 2.

Descriptive Statistics				Communalities		Cronbach’s Alpha	Validity Analysis		
	Mean	Std. Deviation	Analysis N	Initial	Extraction				
**T010103**	.2569	.01382	9430	1.000	.326	.895	Kaiser-Meyer-Olkin Measure of Sampling Adequacy		.904
**T010104**	.0779	.00606	9430	1.000	.859				
**T010105**	.0262	.00635	9430	1.000	.749				
**T010106**	.0384	.00567	9430	1.000	.842				
**T010201**	.0542	.01011	9430	1.000	.784				
**T010202**	.0518	.00900	9430	1.000	.748		Bartlett’s Test of Sphericity	Approx. Chi-Square	114286.447
**T010203**	.1709	.01419	9430	1.000	.613				
**T010205**	.1526	.00809	9430	1.000	.768				
**T010303**	.1147	.00716	9430	1.000	.605			df	120
**T010304**	.1769	.01827	9430	1.000	.690				
**T010305**	.3839	.01730	9430	1.000	.851			Sig.	.000
**T010306**	.2739	.01210	9430	1.000	.667				
**T010307**	.3170	.01568	9430	1.000	.660		Average Communality		.701
**T010308**	.2977	.02124	9430	1.000	.800				
**T010401**	.3824	.01299	9430	1.000	.580		Anti-image Correlations		> 0.5
**T010501**	.0307	.00604	9430	1.000	.677				

### Repeated reliability and validity analysis

A correlation analysis was followed to confirm the strong relationship between the above sixteen items. Correlation analysis suggested that four items (T010103, T010201, T010202, and T010304) should be eliminated due to correlation coefficients (lower than 0.3)—the reliability and validity analysis needed to be re-done for the remaining twelve items. Repeated reliability analysis was conducted. T010308 was eliminated due to dissatisfaction with the value of Cronbach’s Alpha (lower than 0.6).

Following the repeated reliability analysis, the validity analysis was then repeated. The results indicated that the remaining eleven items have strong evidence of meeting the reliability Analysis, and the validity standards of the exploratory research are listed in Table 8. Cronbach’s Alpha was 0.888 and was considered to have acceptable reliability (above 0.8). In the validity test, the ratio of the number of cases to variables was 857:1 (greater than 5:1). KMO was 0.893 and considered to be acceptable (greater than 0.8). Bartlett’s test of sphericity was 0.000 (p<0.001). All communalities after extraction were greater than 0.50. The average communality was 0.766 (greater than 0.6), and all anti-image correlations were satisfied (above 0.5).

**Table 8:** Repeated reliability and validity analysis of gas data in Case Study 2.

Descriptive Statistics				Communalities		Cronbach’s Alpha	Validity Analysis		
	Mean	Std. Deviation	Analysis N	Initial	Extraction				
**T010104**	.0779	.00606	9430	1.000	.871	.888	Kaiser-Meyer-Olkin Measure of Sampling Adequacy		.893
**T010105**	.0262	.00635	9430	1.000	.788				
**T010106**	.0384	.00567	9430	1.000	.853				
**T010203**	.1709	.01419	9430	1.000	.859				
**T010205**	.1526	.00809	9430	1.000	.865		Bartlett’s Test of Sphericity	Approx. Chi-Square	72885.396
**T010303**	.1147	.00716	9430	1.000	.610				
**T010305**	.3839	.01730	9430	1.000	.829				
**T010306**	.2739	.01210	9430	1.000	.765			df	55
**T010307**	.3170	.01568	9430	1.000	.677			Sig.	.000
**T010401**	.3824	.01299	9430	1.000	.624		Average Communality		.766
**T010501**	.0307	.00604	9430	1.000	.683		Anti-image Correlations		> 0.5

Another correlation analysis was then conducted to confirm the relationship between the remaining eleven items (T010104, T010105, T010106, T010203, T010205, T010303, T010305, T010306, T010307, 010401, T010501). Robust correlations (greater than 0.3) existed between every two items at the 0.01 level (S3 Appendix).

### Computing the weights

**Fig 6:**
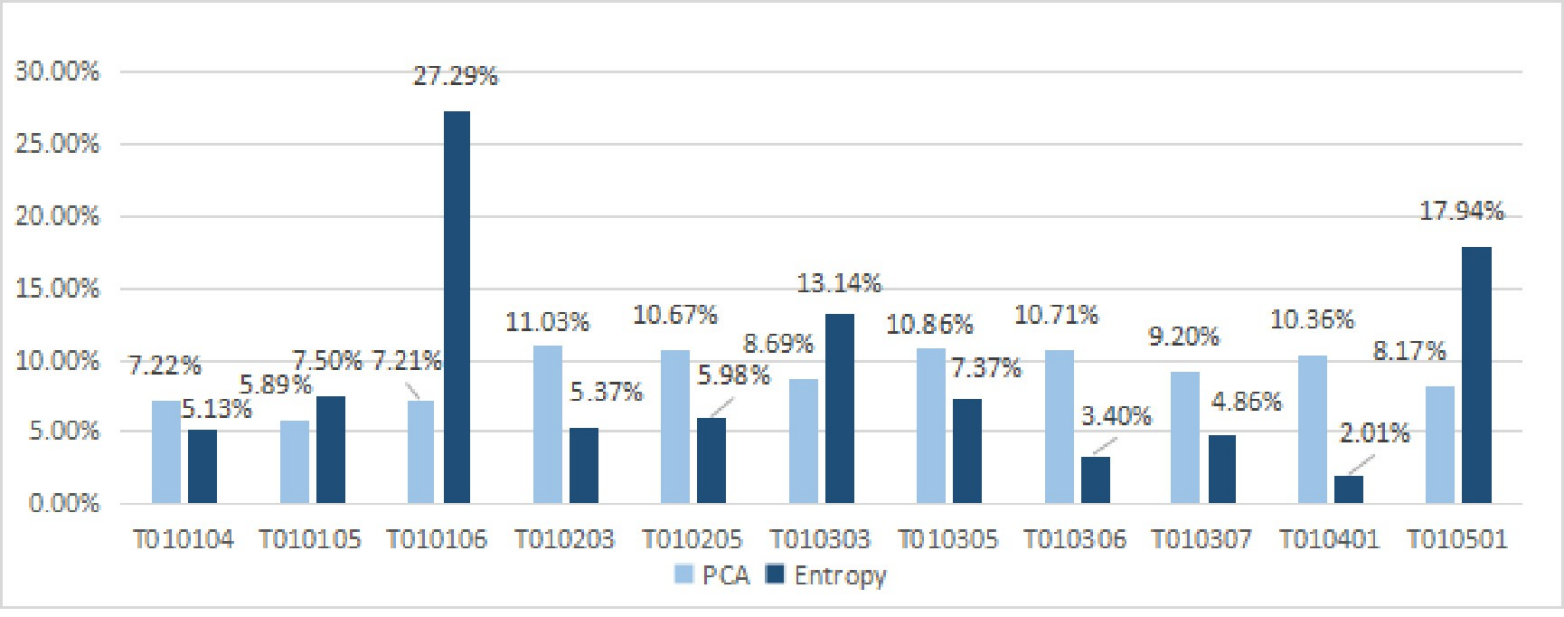
The computed outcomes of weights in Case Study 2.

The PCA and Entropy for Case Study 2 were then computed separately to determine which weighting factor method would be a single responsive approach for Case Study 2. The results indicate that the value of the PCA and Entropy were T010104 (7.22% and 5.13%), T010105 (5.89% and 7.50%), T010106 (7.21% and 27.29%), T010203 (11.03% and 5.37%), T010205 (10.67% and 5.98%), T010303 (8.69% and 13.14%), T010305 (10.86% and 7.37%), T010306 (10.71% and 3.40%), T010307 (9.20% and 4.86%), T010401 (10.36% and 2.01%), and T010501 (8.17% and 17.94%), respectively (Fig 6).

Table 9 describes the mean and standard deviation statistics for the PCA and Entropy methods adopted to Case Study 2. For the weights to T010104, the minimum value is 0.0512; the maximum value is 0.0721; the mean value is 0.0617; the standard deviation is 0.0148. For the weights to T010105, the minimum value is 0.0591; the maximum value is 0.0748; the mean value is 0.067; the standard deviation is 0.0111. For the weights to T010106, the minimum value is 0.0721; the maximum value is 0.2723; the mean value is 0.1722; the standard deviation is 0.1416. For the weights to T010203, the minimum value is 0.053; the maximum value is 0.1108; the mean value is 0.0819; the standard deviation is 0.0409. For the weights to T010205, the minimum value is 0.0598; the maximum value is 0.1075; the mean value is 0.0837; the standard deviation is 0.0337. For the weights to T010303, the minimum value is 0.0872; the maximum value is 0.1322; the mean value is 0.1097; the standard deviation is 0.0318. For the weights to T010305, the minimum value is 0.0741; the maximum value is 0.1079; the mean value is 0.091; the standard deviation is 0.0239. For the weights to T010306, the minimum value is 0.0344; the maximum value is 0.1067; the mean value is 0.706; the standard deviation is 0.0511. For the weights to T010307, the minimum value is 0.487; the maximum value is 0.0915; the mean value is 0.0701; the standard deviation is 0.0303. For the weights to T010401, the minimum value is 0.0202; the maximum value is 0.1035; the mean value is 0.0619; the standard deviation is 0.0589. For the weights to T010501, the minimum value is 0.0816; the maximum value is 0.1792; the mean value is 0.1304; the Standard deviation is 0.069. It means that all data are relatively distributed near the mean value.

**Table 9:** Descriptive on standard deviation to Case Study 2.

	Entropy	PCA	N	Mean	Std. Deviation
**T010104**	5.12%	7.21%	2	6.17%	0.0148
**T010105**	7.48%	5.91%	2	6.70%	0.0111
**T010106**	27.23%	7.21%	2	17.22%	0.1416
**T010203**	5.30%	11.08%	2	8.19%	0.0409
**T010205**	5.98%	10.75%	2	8.37%	0.0337
**T010303**	13.22%	8.72%	2	10.97%	0.0318
**T010305**	7.41%	10.79%	2	9.10%	0.0239
**T010306**	3.44%	10.67%	2	7.06%	0.0511
**T010307**	4.87%	9.15%	2	7.01%	0.0303
**T010401**	2.02%	10.35%	2	6.19%	0.0589
**T010501**	17.92%	8.16%	2	13.04%	0.0566
**Valid N (listwise)**			2		

**Fig 7:**
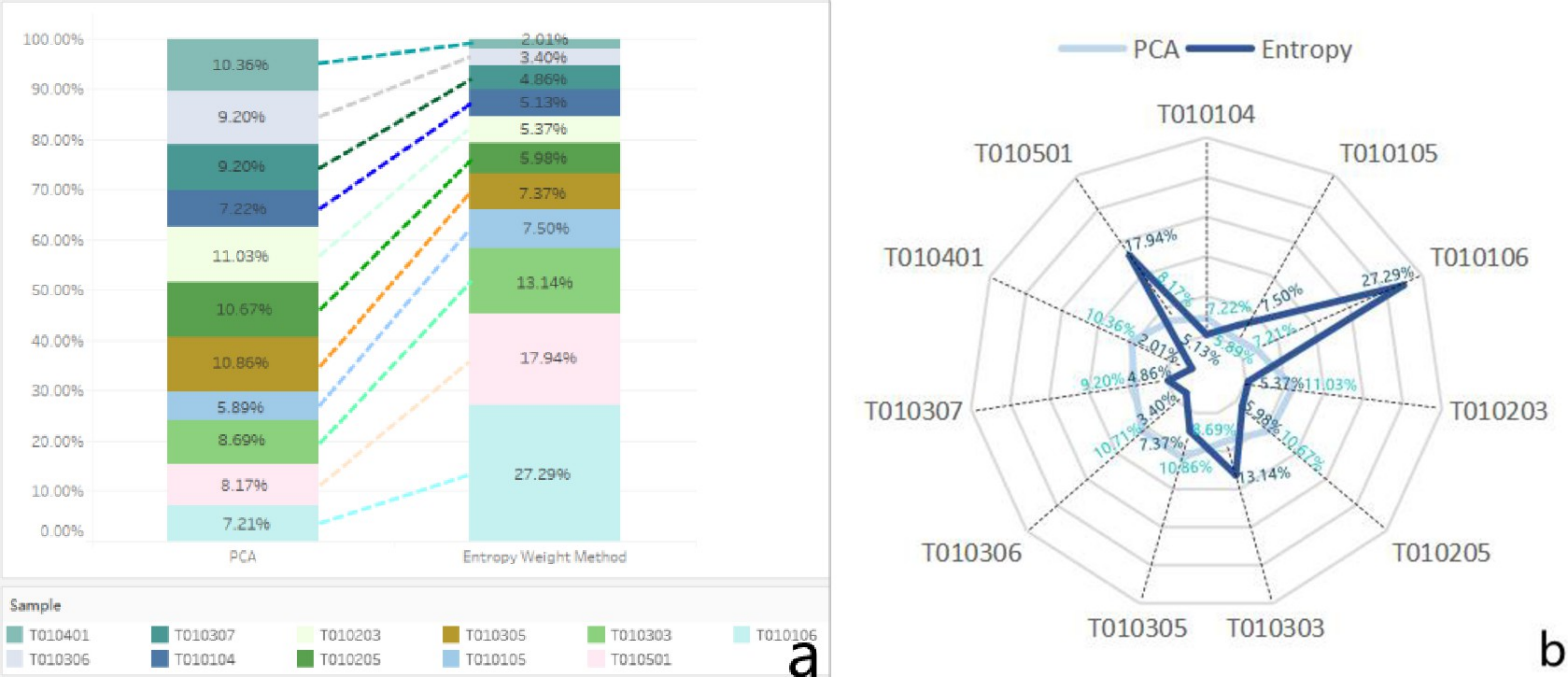
Visualization of comparison of the weighted index in Case Study 2.

Based on the results in Table 9, the comparisons of the weighting methods between the PCA and Entropy methods were demonstrated in Fig 7A. The results were visually set to a weighted indexing measurement with a radar visualization for The PCA (with blue color) and Entropy (with deep blue color) weighted index (Fig 7B).

## Comparative analysis of the PCA and entropy methods

Different MCDM methods may lead to inconsistent results [12, 19]. It is essential to compare results for different MCDM methods [19]. For justifying the above correlation analysis results between the PCA and Entropy method, two comparative analyses were conducted to compare the differences for using the PCA and the Entropy to Case Study 1 and Case Study 2. The comparison will identify whether the different weighting methods may lead to the differentiation of indexing measurements.

### Comparing outcomes between PCA and Entropy in case study 1

**Fig 8:**
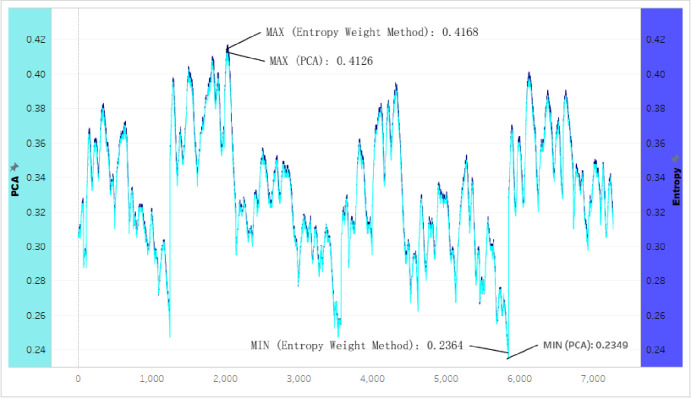
Two Curves of the weighted index between The PCA and entropy in Case Study 1.

Based on the weighted index comparisons in Fig 4 and datasets collected in Case study 1 (S2 Appendix), the final data were computed into two weighted curves (Fig 8). The left axle in Fig 8 stated the values of the Entropy with deep blue color. The right axle stated the values of the PCA with blue color. The two curves were very close.

However, the Entropy calculated the highest value (0.4168) and was higher than the highest (0.4126) calculated by the PCA. The entropy’s lowest value (0.2364) was also higher than the lowest weight (0.2349) estimated by the PCA. The curve of weighted dataset values computed by the PCA method (with blue color) was lower than the curve by the Entropy method (with deep blue color). The PCA appeared to be a more responsive method than Entropy to Case Study 1.

### Comparing outcomes between PCA and entropy in Case Study 2

**Fig 9:**
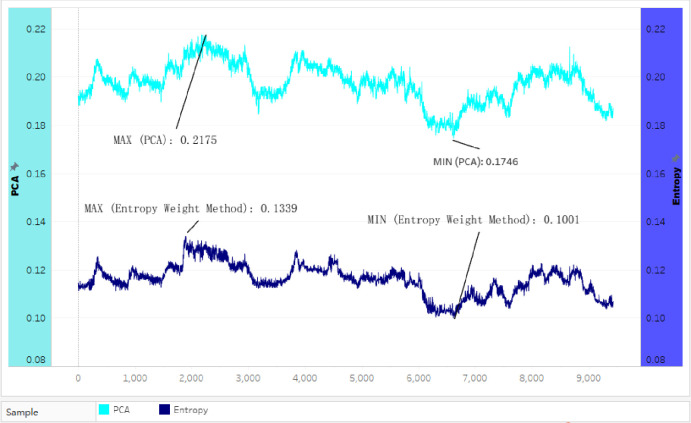
Two curves of the weighted index between the PCA and the entropy in Case Study 2.

Based on the comparisons of the weighted index in Fig 6 and datasets collected in Case study 2 (S3 Appendix), the data were computed into two weighted curves (Fig 9). The left axle in Fig 9 stated the values of the Entropy with deep blue color. The right axle stated the values of the PCA with blue color. There are different values between the two curves.

The curve of weighted dataset values computed by the Entropy method (with deep blue color) was lower than the curve by the PCA method (with blue color). Entropy is more responsive than the PCA to establish a weighted indexing measurement in Case Study 2. The highest value (0.2175) was computed by PCA and was higher than the highest value (0.1339) calculated by Entropy. PCA’s lowest value (0.1746) was also higher than Entropy’s lowest weight (0.1001). Even the PCA’s lowest value (0.1746) was higher than Entropy’s highest (0.1339).

### Comparative analysis of the PCA and entropy methods

This section summarized a comparative analysis of the PCA and Entropy methods to the case studies. The Means and St. Deviations of the two methods to Case Study 1 and Study 2 were described (Table 10).

**Table 10:** Comparative analysis of the PCA and entropy methods.

Dimensions	Case Study 1				Case Study 2			
	Entropy	PCA	Mean	Std. Deviation	Entropy	PCA	Mean	Std. Deviation
**Dim. 1**	32.70%	24.37%	28.54%	0.0059	5.12%	7.21%	6.17%	0.0148
**Dim. 2**	23.82%	24.76%	24.29%	0.0007	7.48%	5.91%	6.70%	0.0111
**Dim. 3**	26.02%	25.51%	25.77%	0.0004	27.23%	7.21%	17.22%	0.1416
**Dim. 4**	17.46%	25.36%	21.41%	0.0056	5.30%	11.08%	8.19%	0.0409
**Dim. 5**					5.98%	10.75%	8.37%	0.0337
**Dim. 6**					13.22%	8.72%	10.97%	0.0318
**Dim. 7**					7.41%	10.79%	9.10%	0.0239
**Dim. 8**					3.44%	10.67%	7.06%	0.0511
**Dim. 9**					4.87%	9.15%	7.01%	0.0303
**Dim. 10**					2.02%	10.35%	6.19%	0.0589
**Dim. 11**					17.92%	8.16%	13.04%	0.0566
**Max-Min**	8.88%	1.14%	7.13%	0.0055	25.21%	5.17%	11.05%	0.1305
**Average of Max-Min**	3.81%	0.29%	1.78%	0.0014	2.29%	0.47%	1.00%	0.0119
**Average**	25.00%	25.00%	25.00%	0.0032	9.09%	9.09%	9.09%	0.0450

For the comparative analysis to Case Study 1, Mean averages are 25.00% as four items (dimensions) were involved. The Entropy method computed the highest weight (32.7%) and the lowest (17.46%). The average of Std. Deviation is 0.0032, which is very small. Therefore, the two weighted curves were very close (Fig 8). The curve of weighted dataset values computed by the PCA method (with blue color) was lower than the curve by the Entropy method (with deep blue color). The PCA appeared to be a more responsive method than Entropy to Case Study 1. Other studies also have different advantages of the PCA method. The PCA method can effectively reduce computational complexity and rapidly select solutions to emergency decision-making in a large dataset group [62]. Notably, the PCA method was sensitive to outliers in the data [63]. Therefore, research suggests it could be a promising alternative for other weighting schemes [64]. However, the PCA method was not developed to identify a subset of variables among many variables that are most predictive of an outcome [34].For the comparative analysis to Case Study 2, Mean averages are 9.09% as eleven items (dimensions) were involved. The Entropy method computed the highest weight (27.23%) and the lowest (2.02%). The average of Std. Deviation is 0.045, which was not small comparing with Case Study 1. Fig 9 visually supported this outcome and demonstrated that the two weighted curves were not close. The curve of weighted dataset values computed by the Entropy method (with deep blue color) was lower than the curve by the PCA method (with blue color). Therefore, Entropy is a more responsive method than the PCA to establish a weighted indexing measurement in Case Study 2. Other studies also believed that the Entropy method allows a quantitative appraisal of effectiveness and advantage/cost responses [23]. It was considered suitable for all the decision-making processes that required weight determination [22]. The main disadvantage of the entropy method for assessing weight is the high sensitivity or hypersensitivity of significance to the entropy values of various criteria [19]. There is also a need to conduct further research to probe the causal reasons why the PCA and Entropy methods were applied to each case and not the other way round.

The difference between maximum and minimum (Max-Min) values by the Entropy and PCA methods in Case Study 1 is 8.88% and 1.14%. The average values of Max-Min are 3.81% and 0.29%. The values of Max-Min by the Entropy and PCA methods in Case Study 2 are 25.21% and 5.17%. The average values of Max-Min are 2.29% and 0.47%. It indicated distinctly that both the values and average values of Max-Min by the Entropy method (8.88% and 1.14%) are higher than the values by the PCA method (3.81% and 0.29%) in Case Study 1. The average values of Max-Min are 2.29% and 0.47%. The values and average values of Max-Min by the Entropy method (25.21% and 5.17%) are higher than the values by the PCA method (2.29% and 0.47%) in Case Study 2. It validated that the Entropy method provides higher accuracy [22]. It may depend on the number of realizations considered: the higher the number, the more accurate the entropy estimate [32].

For Std. Deviation, the values and average values of Max-Min in Case Study 2 (0.1305 and 0.0119) are much higher than those in Case Study 1 (0.0055 and 0.0014). The average value of Std. Deviation in Case Study 2 (0.045) is also much higher than the values in Case Study 1(0.0032). It means that the Entropy values demonstrated higher sensitivity for assessing the weight to the higher dimensions (eleven-dimensions) dataset in Case Study 2 than the lower dimensions (four-dimensions) dataset in Case Study 1. The reason should be considered due to the exponential behavior of the logarithm in the vicinity of 0 [19].

Based on the above comparative analysis of two methods in two case studies, this research indicated that no single method could be adopted as the better option for establishing indexing measurement in all cases. Both the PCA and Entropy methods were relevant to different cases. Different objective methods lead to completely different values in the estimates of the weights of the criteria [19]. Thus, this research confirmed that different MCDM methods produced diverse results to solve the same case [15]. The practical implication suggests that comparative analysis should always be conducted to each case and determine the appropriate weighting method for further establishing weighted indexing measurement in the relevant case. This research recommended that the PCA method was a dimension reduction technique that could be handy for identifying the critical variables or factors and effectively used in hazard, risk, and emergency assessment. Due to the PCA method was sensitive to outliers, this method might also be well-applied for developing predicting and forecasting systems.

The above comparative analysis found that the Entropy method provides higher accuracy than the PCA method. This research also found that the Entropy method demonstrated to assess the weights of the higher dimension dataset (eleven dimensions) in Case Study 2 was higher sensitivity than the lower dimensions (four dimensions) in Case Study 1. As objective methods, both methods ignore decision-makers’ opinions. They determine the weights of criteria based on the information in the decision-making matrix using specific mathematical models [20]. The rationality of all objective methods for evaluating the criteria weights for MCDM tasks is questionable: specific algorithms for objective methods to evaluate the importance of criteria still require further study [19]. Therefore, there is a need to explore a more responsive method for establishing a weighted indexing measurement for warning applications in hazard, risk, and emergency assessments.

## Conclusions, recommendations, and need for further research

The literature found that the PCA method was the predominant weighting method adopted in China’s applications, and the Entropy method was the second most widely adopted weighting method. This paper aimed to determine which method between the PCA and Entropy was better for establishing a responsive weighted indexing measurement. The selected method would be used to build robust monitoring or warning systems for improving coal mining safety.

Both the PCA and Entropy methods were compared using two case studies. Based on the comparative analysis of the PCA and Entropy methods in two case studies, this research confirmed that different objective MCDM methods produced completely different weights to the same case. The comparative analysis indicated that no single approach between the PCA and Entropy methods could be adopted as better for establishing indexing measurement. The PCA and Entropy methods were adopted for different cases in case study mine. For the comparative analysis of Case Study 1, the PCA appeared to be a more responsive method than Entropy. For the comparative analysis of Case Study 2, Entropy is a more responsive method than the PCA to establish a weighted indexing measurement. As a result, both methods were adopted for different cases in the case study mine and finally deployed for user acceptance testing on 5 November 2020.

The main limitation is the correlational research method itself. The correlational research does not allow for identifying causal relationships [65]. Another limitation is related to both the PCA and Entropy methods themselves that they are limited to ignoring the opinions from domain experts and decision-makers. The third limitation is that this research did not confirm any single method to adopt as the best option to all cases. One more limitation is the limited number of cases to be acknowledged in this research.

The findings and suggestions were provided as further scopes for further research. This research indicated that no single method could be adopted as the better option for establishing indexing measurement in all cases. The practical implication suggests that comparative analysis should always be conducted on each case and determine the appropriate weighting method to the relevant case. This research recommended that the PCA method was a dimension reduction technique that could be handy for identifying the critical variables or factors and effectively used in hazard, risk, and emergency assessment. The PCA method might also be well-applied for developing predicting and forecasting systems as it was sensitive to outliers. The Entropy method might be suitable for all the cases requiring the MCDM. There is also a need to conduct further research to probe the causal reasons why the PCA and Entropy methods were applied to each case and not the other way round. This research found that the Entropy method provides higher accuracy than the PCA method. This research also found that the Entropy method demonstrated to assess the weights of the higher dimension dataset was higher sensitivity than the lower dimensions. Finally, the comprehensive analysis indicates a need to explore a more responsive method for establishing a weighted indexing measuremfent for warning applications in hazard, risk, and emergency assessments.

## Supporting information

S1 Appendix Code for running the entropy algorithm. (DOC)

S2 Appendix Data collected in Case Study 1. (DOC)

S3 Appendix Correlation analysis in Case Study 2. (DOC)

S4 Appendix Data collected in Case Study 2. (DOC)
