# Correction: A comparative analysis of the principal component analysis and entropy weight methods to establish the indexing measurement

**DOI:** 10.1371/journal.pone.0314513

**Published:** 2024-11-21

**Authors:** The PLOS ONE Staff

There is an error in affiliation 2 for authors Robert M. X. Wu, Zhongwu Zhang, and Jianfeng Fan. The correct affiliation 2 is: Shanxi Normal University, Taiyuan, China.

The publisher apologizes for the error.

## References

[1] Wu RMX, Zhang Z, Yan W, Fan J, Gou J, Liu B, et al. (2022) A comparative analysis of the principal component analysis and entropy weight methods to establish the indexing measurement. PLOS ONE 17(1): e0262261. doi: 10.1371/journal.pone.0262261 35085274 PMC8802816

