# Supplementary figures and images for: A comparative analysis of the principal component analysis and entropy weight methods to establish the indexing measurement
Source: PLoS One. 2022 Jan 27;17(1):e0262261. doi: 10.1371/journal.pone.0262261 (PMC8802816; doi:10.1371/journal.pone.0262261)

Appendix 2: Data Collected in Case Study 1


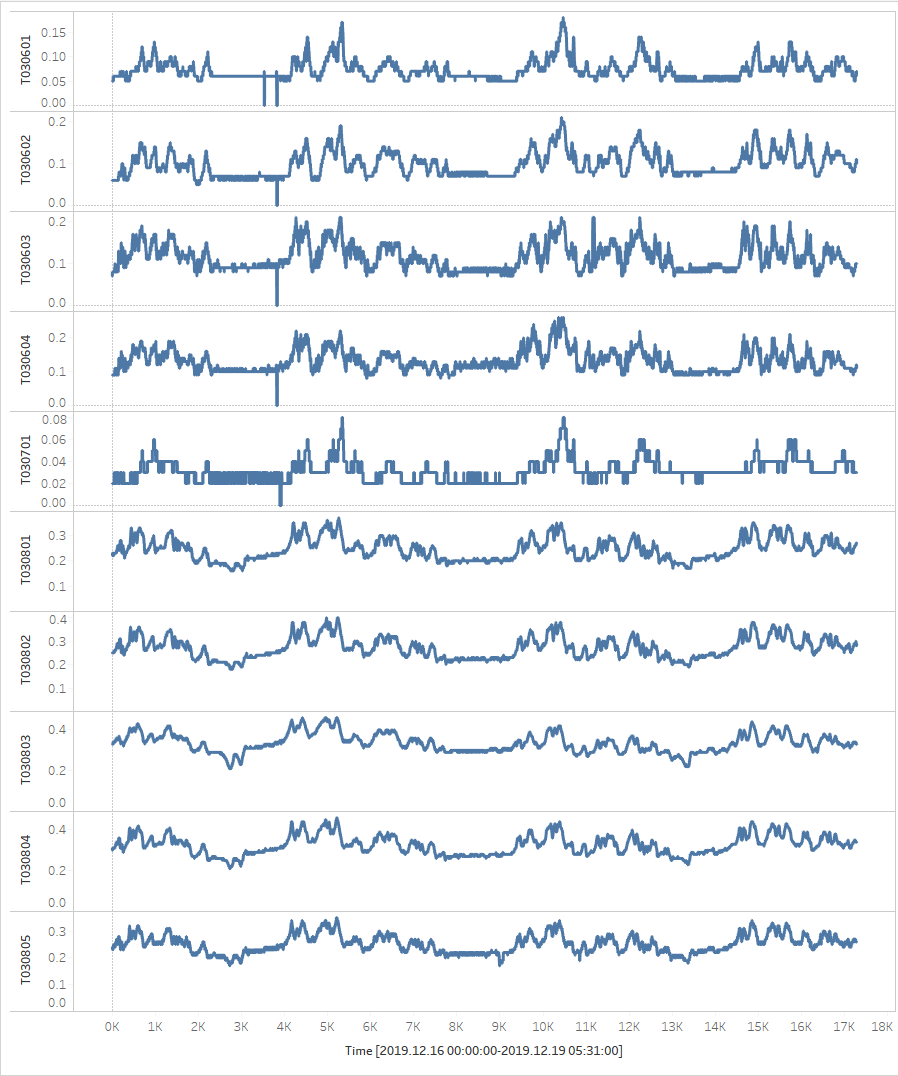

Supplement: S2 Appendix — (DOC) [file pone.0262261.s002.doc]

Appendix 4: Data Collected in Case Study 2


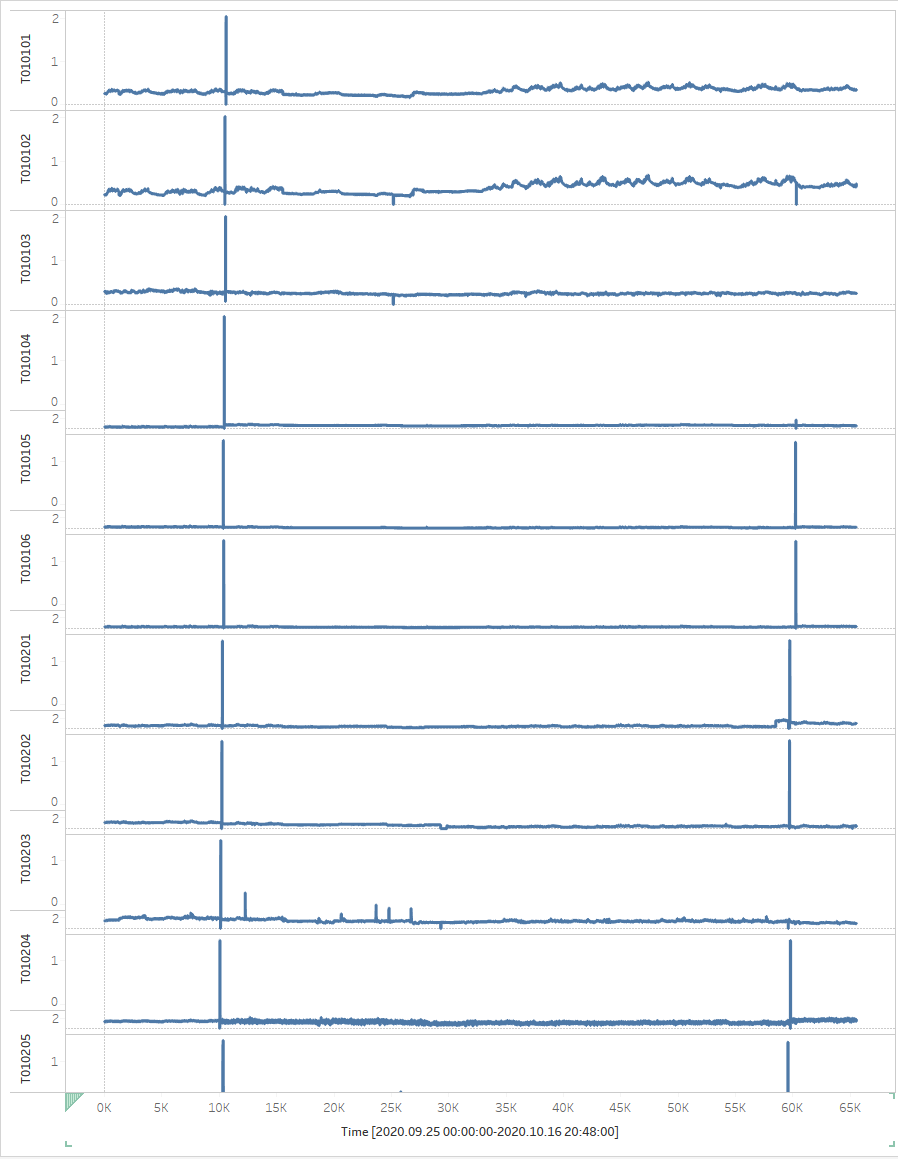


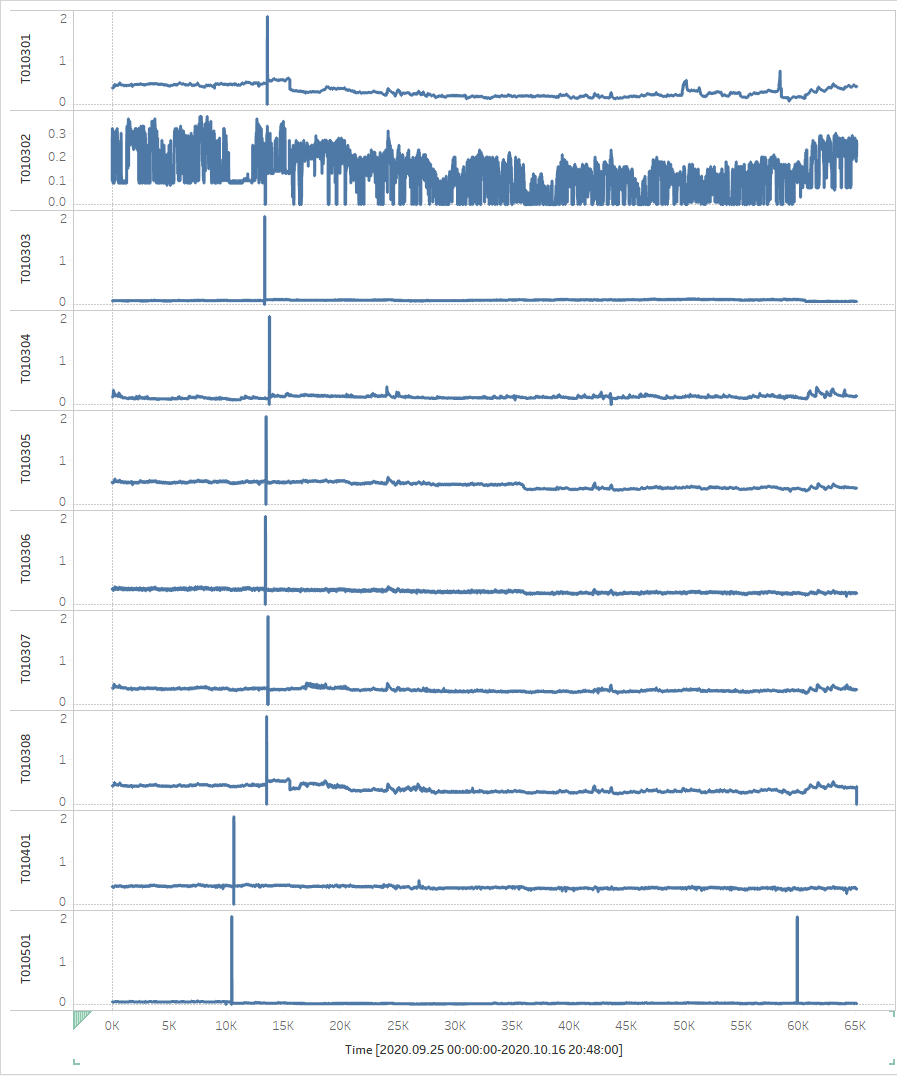

Supplement: S4 Appendix — (DOC) [file pone.0262261.s004.doc]
