# Supplementary material for: A comparative analysis of the principal component analysis and entropy weight methods to establish the indexing measurement
Source: PLoS One. 2022 Jan 27;17(1):e0262261. doi: 10.1371/journal.pone.0262261 (PMC8802816; doi:10.1371/journal.pone.0262261)
Supplement: S3 Appendix — (DOC) [file pone.0262261.s003.doc]

Appendix 3: Correlation Analysis in Case Study 2

| **Correlations** | | | | | | | | | | | | |
| --- | --- | --- | --- | --- | --- | --- | --- | --- | --- | --- | --- | --- |
|  |  | **T010104** | **T010105** | **T010106** | **T010203** | **T010205** | **T010303** | **T010305** | **T010306** | **T010307** | **T010401** | **T010501** |
| **T010104** | Pearson Correlation | 1 | .767** | .861** | .429** | .467** | .633** | .409** | .453** | .527** | .542** | .711** |
|  | Sig. (2-tailed) |  | 0 | 0 | 0 | 0 | 0 | 0 | 0 | 0 | 0 | 0 |
|  | Sum of Squares and Cross-products | 0.347 | 0.279 | 0.28 | 0.347 | 0.216 | 0.26 | 0.406 | 0.315 | 0.474 | 0.405 | 0.246 |
|  | Covariance | 0 | 0 | 0 | 0 | 0 | 0 | 0 | 0 | 0 | 0 | 0 |
|  | N | 9444 | 9444 | 9444 | 9444 | 9444 | 9444 | 9444 | 9444 | 9444 | 9444 | 9444 |
| **T010105** | Pearson Correlation | .767** | 1 | .750** | .371** | .443** | .573** | .335** | .373** | .449** | .450** | .621** |
|  | Sig. (2-tailed) | 0 |  | 0 | 0 | 0 | 0 | 0 | 0 | 0 | 0 | 0 |
|  | Sum of Squares and Cross-products | 0.279 | 0.381 | 0.255 | 0.315 | 0.215 | 0.247 | 0.348 | 0.272 | 0.424 | 0.352 | 0.225 |
|  | Covariance | 0 | 0 | 0 | 0 | 0 | 0 | 0 | 0 | 0 | 0 | 0 |
|  | N | 9444 | 9444 | 9444 | 9444 | 9444 | 9444 | 9444 | 9444 | 9444 | 9444 | 9444 |
| **T010106** | Pearson Correlation | .861** | .750** | 1 | .428** | .466** | .611** | .440** | .436** | .537** | .517** | .711** |
|  | Sig. (2-tailed) | 0 | 0 |  | 0 | 0 | 0 | 0 | 0 | 0 | 0 | 0 |
|  | Sum of Squares and Cross-products | 0.28 | 0.255 | 0.304 | 0.324 | 0.202 | 0.235 | 0.409 | 0.284 | 0.452 | 0.361 | 0.23 |
|  | Covariance | 0 | 0 | 0 | 0 | 0 | 0 | 0 | 0 | 0 | 0 | 0 |
|  | N | 9444 | 9444 | 9444 | 9444 | 9444 | 9444 | 9444 | 9444 | 9444 | 9444 | 9444 |
| **T010203** | Pearson Correlation | .429** | .371** | .428** | 1 | .738** | .446** | .394** | .367** | .388** | .449** | .472** |
|  | Sig. (2-tailed) | 0 | 0 | 0 |  | 0 | 0 | 0 | 0 | 0 | 0 | 0 |
|  | Sum of Squares and Cross-products | 0.347 | 0.315 | 0.324 | 1.889 | 0.797 | 0.427 | 0.912 | 0.596 | 0.813 | 0.781 | 0.38 |
|  | Covariance | 0 | 0 | 0 | 0 | 0 | 0 | 0 | 0 | 0 | 0 | 0 |
|  | N | 9444 | 9444 | 9444 | 9444 | 9444 | 9444 | 9444 | 9444 | 9444 | 9444 | 9444 |
| **T010205** | Pearson Correlation | .467** | .443** | .466** | .738** | 1 | .476** | .364** | .396** | .322** | .451** | .466** |
|  | Sig. (2-tailed) | 0 | 0 | 0 | 0 |  | 0 | 0 | 0 | 0 | 0 | 0 |
|  | Sum of Squares and Cross-products | 0.216 | 0.215 | 0.202 | 0.797 | 0.619 | 0.261 | 0.483 | 0.368 | 0.387 | 0.45 | 0.215 |
|  | Covariance | 0 | 0 | 0 | 0 | 0 | 0 | 0 | 0 | 0 | 0 | 0 |
|  | N | 9444 | 9444 | 9444 | 9444 | 9444 | 9444 | 9444 | 9444 | 9444 | 9444 | 9444 |
| **T010303** | Pearson Correlation | .633** | .573** | .611** | .446** | .476** | 1 | .419** | .507** | .557** | .577** | .564** |
|  | Sig. (2-tailed) | 0 | 0 | 0 | 0 | 0 |  | 0 | 0 | 0 | 0 | 0 |
|  | Sum of Squares and Cross-products | 0.26 | 0.247 | 0.235 | 0.427 | 0.261 | 0.486 | 0.493 | 0.417 | 0.593 | 0.51 | 0.231 |
|  | Covariance | 0 | 0 | 0 | 0 | 0 | 0 | 0 | 0 | 0 | 0 | 0 |
|  | N | 9444 | 9444 | 9444 | 9444 | 9444 | 9444 | 9444 | 9444 | 9444 | 9444 | 9444 |
| **T010305** | Pearson Correlation | .409** | .335** | .440** | .394** | .364** | .419** | 1 | .720** | .682** | .573** | .484** |
|  | Sig. (2-tailed) | 0 | 0 | 0 | 0 | 0 | 0 |  | 0 | 0 | 0 | 0 |
|  | Sum of Squares and Cross-products | 0.406 | 0.348 | 0.409 | 0.912 | 0.483 | 0.493 | 2.839 | 1.433 | 1.755 | 1.222 | 0.479 |
|  | Covariance | 0 | 0 | 0 | 0 | 0 | 0 | 0 | 0 | 0 | 0 | 0 |
|  | N | 9444 | 9444 | 9444 | 9444 | 9444 | 9444 | 9444 | 9444 | 9444 | 9444 | 9444 |
| **T010306** | Pearson Correlation | .453** | .373** | .436** | .367** | .396** | .507** | .720** | 1 | .549** | .623** | .475** |
|  | Sig. (2-tailed) | 0 | 0 | 0 | 0 | 0 | 0 | 0 |  | 0 | 0 | 0 |
|  | Sum of Squares and Cross-products | 0.315 | 0.272 | 0.284 | 0.596 | 0.368 | 0.417 | 1.433 | 1.394 | 0.99 | 0.932 | 0.329 |
|  | Covariance | 0 | 0 | 0 | 0 | 0 | 0 | 0 | 0 | 0 | 0 | 0 |
|  | N | 9444 | 9444 | 9444 | 9444 | 9444 | 9444 | 9444 | 9444 | 9444 | 9444 | 9444 |
| **T010307** | Pearson Correlation | .527** | .449** | .537** | .388** | .322** | .557** | .682** | .549** | 1 | .492** | .508** |
|  | Sig. (2-tailed) | 0 | 0 | 0 | 0 | 0 | 0 | 0 | 0 |  | 0 | 0 |
|  | Sum of Squares and Cross-products | 0.474 | 0.424 | 0.452 | 0.813 | 0.387 | 0.593 | 1.755 | 0.99 | 2.33 | 0.951 | 0.455 |
|  | Covariance | 0 | 0 | 0 | 0 | 0 | 0 | 0 | 0 | 0 | 0 | 0 |
|  | N | 9444 | 9444 | 9444 | 9444 | 9444 | 9444 | 9444 | 9444 | 9444 | 9444 | 9444 |
| **T010401** | Pearson Correlation | .542** | .450** | .517** | .449** | .451** | .577** | .573** | .623** | .492** | 1 | .510** |
|  | Sig. (2-tailed) | 0 | 0 | 0 | 0 | 0 | 0 | 0 | 0 | 0 |  | 0 |
|  | Sum of Squares and Cross-products | 0.405 | 0.352 | 0.361 | 0.781 | 0.45 | 0.51 | 1.222 | 0.932 | 0.951 | 1.604 | 0.379 |
|  | Covariance | 0 | 0 | 0 | 0 | 0 | 0 | 0 | 0 | 0 | 0 | 0 |
|  | N | 9444 | 9444 | 9444 | 9444 | 9444 | 9444 | 9444 | 9444 | 9444 | 9444 | 9444 |
| **T010501** | Pearson Correlation | .711** | .621** | .711** | .472** | .466** | .564** | .484** | .475** | .508** | .510** | 1 |
|  | Sig. (2-tailed) | 0 | 0 | 0 | 0 | 0 | 0 | 0 | 0 | 0 | 0 |  |
|  | Sum of Squares and Cross-products | 0.246 | 0.225 | 0.23 | 0.38 | 0.215 | 0.231 | 0.479 | 0.329 | 0.455 | 0.379 | 0.344 |
|  | Covariance | 0 | 0 | 0 | 0 | 0 | 0 | 0 | 0 | 0 | 0 | 0 |
|  | N | 9444 | 9444 | 9444 | 9444 | 9444 | 9444 | 9444 | 9444 | 9444 | 9444 | 9444 |
| ** Correlation is significant at the 0.01 level (2-tailed). | | | | | | | | | | | | |
